# Protective effect of ghrelin on intestinal I/R injury in rats

**DOI:** 10.1515/med-2022-0520

**Published:** 2022-07-20

**Authors:** Meng Jiang, Shengxia Wan, Xiaoyong Dai, Youwen Ye, Wei Hua, Guoguang Ma, Xiufeng Pang, Huanhuan Wang, Bin Shi

**Affiliations:** Department of Emergency Intensive Care Unit, Yangpu Hospital, Tongji University, Shanghai 200090, China; Department of Neurology, The Fourth Affiliated Hospital of Jiangsu University, Zhenjiang 212000, China; Department of Critical Care Medicine, Zhongshan Hospital, Fudan University, Shanghai 200032, China

**Keywords:** ghrelin, intestinal ischemia–reperfusion injury, NOD2/Beclin-1

## Abstract

This study aimed to investigate whether ghrelin affected the autophagy and inflammatory response of intestinal intraepithelial lymphocytes (IELs) by regulating the NOD2/Beclin-1 pathway in an intestinal ischemia–reperfusion (I/R) injury model. Twenty hours after implementing the intestinal I/R injury rat model, the small intestine and both lungs were collected for histological analysis. The morphological changes in the intestinal mucosa epithelium and lung tissues were evaluated using hematoxylin-eosin staining. The activity of autophagic vacuoles and organ injury were evaluated using electron microscopy. The cytokine levels (IL-10 and TNF-α) in IEL cells and lung tissue were determined using enzyme-linked immunosorbent assay. RT-qPCR and western blot assays were conducted to check the NOD2, Beclin-1, and ATG16 levels. Ghrelin relieved the I/R-induced destruction of the intestinal mucosa epithelium and lung tissues. Moreover, ghrelin enhanced autophagy in the intestinal epithelium and lungs of I/R rats. In addition, the levels of autophagy-associated proteins (Beclin-1, ATG16, and NOD2) were higher in the ghrelin treatment group than in rats with I/R. Ghrelin reduced significantly the IL-10 and TNF-α levels. However, these changes were reversed by the NOD2 antagonist. In conclusion, ghrelin may relieve I/R-induced acute intestinal mucosal damage, autophagy disorder, and inflammatory response in IELs by regulating the NOD2/Beclin-1 pathway.

## 1 Introduction

Intestinal ischemia–reperfusion (I/R) injury, a serious abdominal emergency with high mortality rate, is commonly found in patients affected by trauma, burns, and hemorrhagic shock as well as organ transplantation [1,2]. Reperfusion may induce remote organ damages and dysfunction by producing various pro-inflammatory cytokines and activating intestinal immune cells, thereby not only resulting in intestinal motility injury but also resulting in immune imbalance [3,4]. However, the latent mechanism of immune response following I/R remains poorly understood [5].

Autophagy is a self-protection process of cells, which degrades and recycles organelles and proteins through the autophagy lysosomal process; this process is vital for maintaining the energy balance and cell homeostasis during starvation [6,7]. Several studies have suggested that I/R injury may result in autophagic dysfunction [8], the abnormal activation of intestinal intraepithelial lymphocyte (IEL) [9], and intestinal, immune, and distant organ damage [10]. Many genes have been reported to mediate autophagy in mammals. For example, in intestinal epithelial cells, impaired autophagy causes intestinal inflammation-related damage and adhesion dysfunction [11]. Therefore, inhibiting autophagy-related injury may provide a new therapeutic approach for the protection of intestinal barrier function in I/R injury.

NOD2, a family member of NOD-like receptors, is identified as a vital intracellular sensor in autophagy and intestinal cell immune balance. Our previous studies have confirmed that NOD2-RIP2 is involved in the small intestine damage regulated by MDP [12]. Moreover, Wu et al. have confirmed that muramyl dipeptide enhances thermal injury-induced autophagy and the inflammatory cytokine response of lungs by the activation of the NOD2/RICK signaling pathway in rats [13]. NOD2 is highly expressed in intestinal epithelial cells and immune cells, including dendritic [14], Paneth [15], and lymphoid cells [16], which play important roles in autophagy, the regulation of the intestinal immune function, and the balance of the intestinal inflammatory response. Previous studies demonstrated that the NOD2/Beclin-1 pathway is involved in autophagy under intestinal stress, which closely related to the amplification of the inflammatory response cascade [17,18]. However, the relationship between IEL autophagy and the NOD2/Beclin-1 pathway in intestinal I/R injury remains elusive.

Ghrelin, an orexigenic hormone, was first identified as an endogenous ligand for GHSR-1a. Ghrelin can regulate autophagy, anti-inflammation, and anti-oxidation as well as downregulate stress [19,20]. In addition, ghrelin has a natural regulatory effect on many diseases, including sepsis [21], chronic inflammatory bowel disease, and intestinal injury [22]. Our previous study showed that ghrelin not only promotes autophagy-related genes in septic intestinal epithelial cells [23] but also regulates the level of NOD2 mRNA and improves lung injury in septic rats [24]. Studies have shown that ghrelin regulates autophagy in IEL cells after intestinal I/R injury and inflammation after IEL activation. However, whether ghrelin can affect IEL autophagy and immune activity through the NOD2/Beclin-1 pathway, especially in the small intestine, needs to be further researched and confirmed.

The aforementioned studies led us to hypothesize that intestinal I/R injury would have a significant effect on IEL autophagy, which would lead to epithelial dysfunction after intestinal I/R. Our research was designed to: (1) investigate the relationship among IEL autophagy, NOD2/Beclin-1, and intestinal and lung injury after I/R injury; (2) evaluate the regulatory effect of ghrelin on IEL autophagy, abnormal intestinal immune activation, and intestinal inflammation after intestinal I/R; and (3) examine the roles of NOD2/Beclin-1 in ghrelin regulatory functions in intestinal I/R. Our findings revealed the protective role of ghrelin in the intestinal I/R injury model by regulating organ damage, autophagy disorder, and the inflammatory response via the NOD2/Beclin-1 pathway. These findings provide a theoretical and therapeutic basis for intestinal I/R injury.

## 2 Methods

### 2.1 Animal model of gut I/R

Male Sprague‒Dawley rats (275–325 g) were provided by the animal experimental center of Tongji University Medical College. The rats were divided into four groups (*n* = 10): sham, intestinal I/R, I/R + ghrelin, and I/R + ghrelin + NOD2 antagonist groups. The rats were kept in a single ventilated cage and allowed to eat and drink enough water in a sterile standard experimental environment. Before ischemia induction, the rats were fasted overnight but allowed to drink freely. Ketamine (75 mg/kg) and midazolam (5 mg/kg) were injected intraperitoneally to anesthetize the animals. After anesthetizing the animals, a middle line incision was made in the lower xiphoid abdomen to separate the superior mesenteric artery. The root of the superior mesenteric artery was clamped with a noninvasive microvascular clamp to restore the blood supply after 75 min of intestinal ischemia. Then I/R models were implemented after reperfusion. All experiments in the present study were approved by the Animal Ethics Committee of Yangpu Hospital, Tongji University, and were conducted in line with the guidelines for the Care and Use of Laboratory Animals of the National Institutes of Health.

### 2.2 Intravenous administration of ghrelin and NOD2 antagonist

Thirty minutes after the administration of an intravenous injection of 2.5 μm camotaxel, I/R was performed after laparotomy. Immediately after removing the microvascular clip, ghrelin (2 nmol; Phoenix Pharmaceuticals, CA, USA) or a vehicle (100 μM normal saline) was administered intravenously for 20 h (8 μL/h) through a 200-μL micropump until the sampling site was killed. Peripheral blood and double lobes were collected from small intestine blood and tissue samples for subsequent studies.

### 2.3 IEL isolation

The small intestine of rats was removed, rinsed, and placed in a culture medium (RPMI-1640, with 10% fetal bovine serum). Then, the intestine was cut into 3–5 cm fragments, washed with extraction buffer, and hatched in buffer with continuous stirring for 30 min. Next, the supernatant was filtered fleetly via a glass wool column and centrifuged at 400×*g* for 5 min. Then, the pellets were purified in 40% Percoll (GE Healthcare Biosciences) and re-suspended in RPMI-1640 culture medium. The cells were cultured in six-well plates and frozen stored for subsequent experiments.

### 2.4 Enzyme-linked immunosorbent assay (ELISA)

After the treatment, the IEL cells or pulmonary tissue were collected and centrifuged for 10 min at 400×*g*. Then, the inflammatory cytokines (IL-10 and TNF-α) in IEL cells and pulmonary tissue were quantified using ELISA kits (BioLegend, Inc., CA, USA) according to the manufacturer’s instructions. The optical density value at 450 nm was determined on a Multiscan Spectrum (Bio-Tek, China) following the manufacturer’s instructions.

### 2.5 Electron microscopy

Intestinal tissues were fixed using 2% glutaraldehyde, washed using 0.1 M phosphate buffer, and cut into ultrathin slices. Then, the tissue slices were dehydrated and stained. We used electron microscopy (Nikon Corporation, China) to evaluate the activity of autophagic vacuoles and organ injury. The autophagosome density was measured using an image software (Experimental Centre of Fudan University, China).

### 2.6 Histological analysis

Small intestine tissue and lungs were harvested, fixed with paraformaldehyde, dehydrated, and embedded in paraffin. The sections were stained with hematoxylin and eosin, and the morphological changes in the stained sections were observed under an optical microscope (CX41, Tokyo, Japan). The histological score was evaluated following the previous study.

### 2.7 qPCR analysis

Total RNA from IEL cells was extracted using a TRIzol reagent (Invitrogen, USA) according to the manufacturer’s protocol. Then, the RNA was reverse-transcribed to cDNA using a cDNA Synthesis Kit (TaKaRa, Beijing, China), and qRT-PCR was performed using an ABI 7500 Real-Time PCR System (Applied Biosystems) with SYBR Premix Ex TaqTM II (TaKaRa). The following primers were used:

β-actin-forward, 5ʹ-CTTCTTTGCAGCTCCTTCGTT-3ʹ;

reverse, 5ʹ-AGGAGTCCTTCTGACCCATTC-3ʹ;

Atg16-forward, 5ʹ-ATGCGCGGATTGTCTCAGGG-3ʹ;

reverse, 5ʹ-GTCCACTCATTACACATTGCTCT-3ʹ;

Beclin-1-forward, 5ʹ-GGCTGAGAGACTGGATCAGG-3ʹ;

reverse, 5ʹ-CTGCGTCTGGGCATAACG-3ʹ;

NOD2-forward, 5ʹ-ATGAGATCCAGCTGTTGTGACATGTG-3ʹ;

reverse, 5ʹ-CTACAGTCCACTCACAAACGGAGAC-3ʹ;

The expression of target genes was calculated using the 2^−ΔΔCt^ method.

### 2.8 Western blot assay

Total proteins from IEL cells were lysed using a protein buffer (Beyotime, China). Protein concentration was determined using the BCA protein quantitative kits (Sigma, USA). The extracted protein samples were separated by 7.5% sodium dodecyl sulfate-polyacrylamide gel electrophoresis and transferred to polyvinylidene fluoride membranes (Millipore, USA). The membranes were then blocked with 7.5% fat-free milk overnight and incubated with the primary antibodies against NOD2, Beclin 1, Atg16, and GAPDH (at a 1:1,500 dilution; Abcam, USA) at room temperature for 1 h. After washing with tris-buffered saline with Tween-20, the membranes were cultured along with secondary antibodies at room temperature for 1 h (1:5,000; Abcam). Finally, the protein bands were analyzed using the enhanced chemiluminescence detection kits (BestBio, China) according to the manufacturer’s instructions.

### 2.9 Statistical analysis

IBM SPSS Statistics for Windows, version 21.0 (IBM Corp., Armonk, NY, USA) was used for statistical analysis. The statistical values were expressed as mean ± standard deviation from at least three independent experiments. Measurement differences among groups were analyzed using one-way analysis of variance and Student’s *t*-test. Data from the analysis of histopathology were compared using the Kruskal–Wallis analysis of variance on ranks test followed by the Dunnett T3 post hoc test for multiple comparisons. *P < 0.05 and **P < 0.01 denoted significant differences.

## 3 Results

### 3.1 Ghrelin improved microscopic intestinal damage following intestinal I/R injury

**Figure 1:**
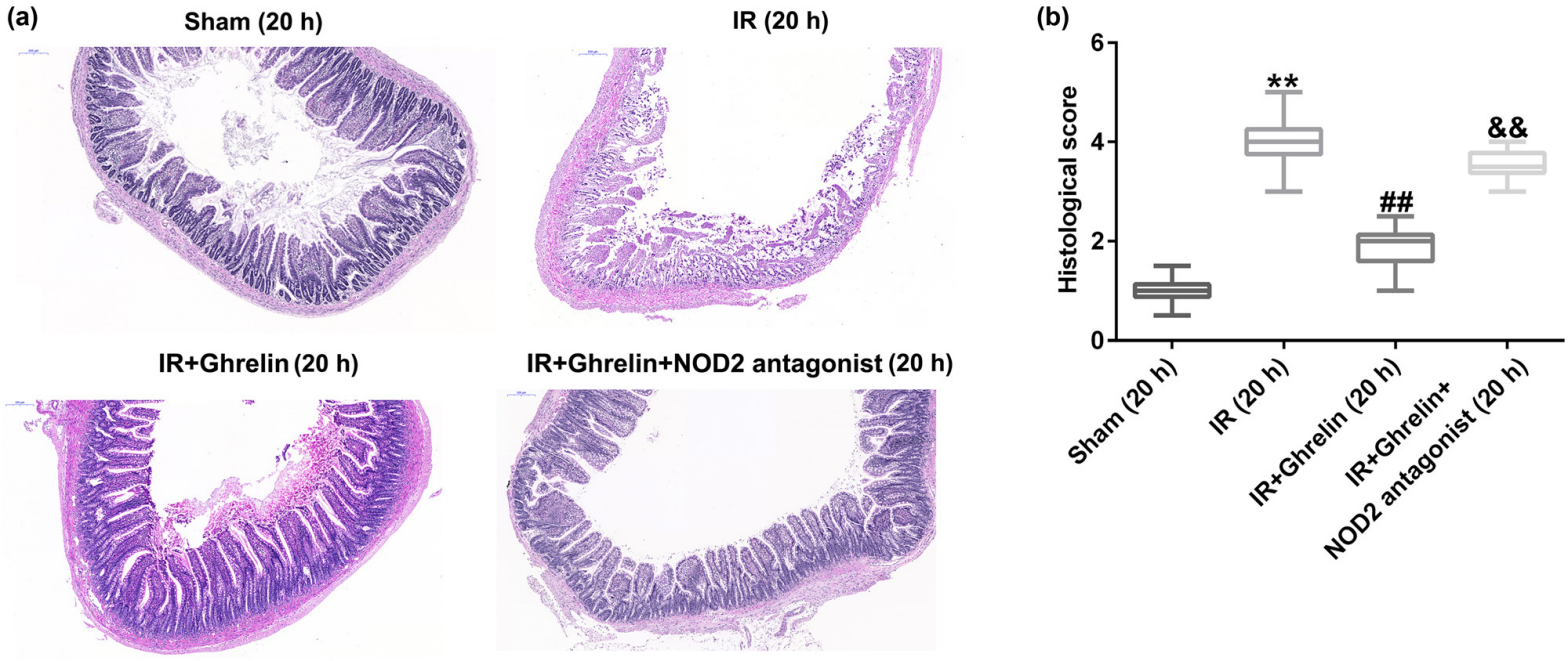
NOD2 antagonist reversed the effects of ghrelin on small bowel tissue injury. Rats were euthanized at either 4 or 20 h after treatment, and their small intestine tissue samples were obtained. (a) Representative intestinal sections from rats of different groups were stained with hematoxylin and eosin. The epithelial structure was clearly demarcated in the sham group. More serious mucosal damage was found in the I/R group than in the sham group. Ghrelin relieved mucosal injury compared to the I/R group. The NOD2 antagonist aggravated mucosal injury in the I/R group. (b) The histological score was determined. Data were expressed as mean ± SD, *n* = 6; **P < 0.01 vs sham; ^##^ *P* < 0.01 vs IR; and *P* < 0.01 vs IR + ghrelin.

In this study, we established intestinal I/R injury models and treated Sprague–Dawley rats with ghrelin or a NOD2 antagonist. Then, the animals were euthanized 20 h after treatment to collect their small intestines. Intestinal sections from rats of different groups were stained with hematoxylin and eosin. As illustrated in Figure 1a, the epithelial structure was clearly demarcated in the sham group. Moreover, we observed more serious mucosal injury in the I/R group than in the sham group. However, compared to the I/R group, the mucosal injury was ameliorated by ghrelin, and this effect was abolished by the NOD2 antagonist (Figure 1a). Figure 1b shows that the reduced histological score in IR + ghrelin group was significantly reversed by NOD2 antagonist. These findings revealed that ghrelin attenuated microscopic intestinal damage following intestinal I/R injury by regulating NOD2.

### 3.2 Ghrelin promoted autophagy in the small intestinal epithelial cells of rats with intestinal I/R injury

**Figure 2:**
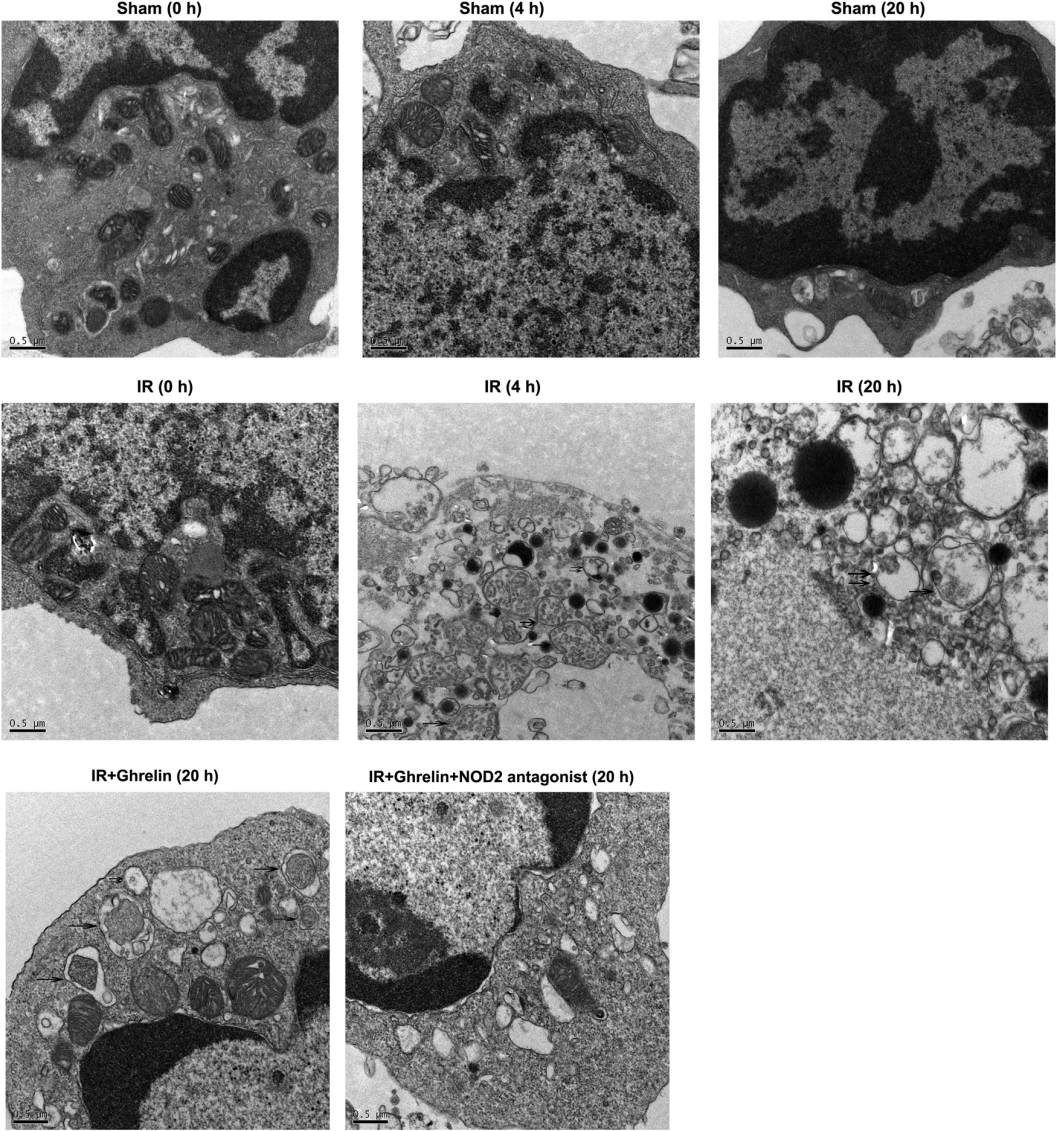
NOD2 antagonist inhibited the phagocytic functions of ghrelin in the intestinal epithelium of rats. Representative electron microscopy images of the intestinal epithelium. Double arrowheads indicated autophagosomes and single arrowheads indicated autolysosomes containing organelles. *n* = 6.

**Figure 3:**
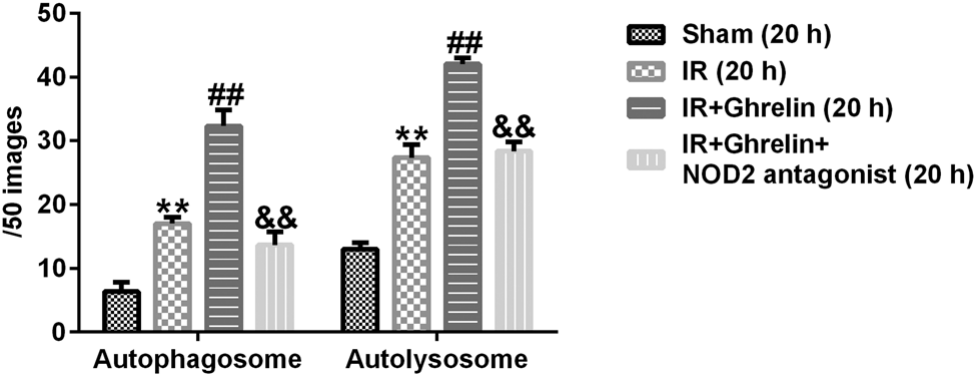
The number of autophagosomes and autolysosomes in the intestinal epithelium of rats. The number of autophagosomes and autolysosomes in the IR group, the ghrelin-treated group, the ghrelin and NOD2 antagonist treatment group, and the sham group at 20 h after I/R. Data were expressed as mean ± SD, *n* = 6; **P < 0.01 vs. sham; ^##^ *P* < 0.01 vs. IR; and *P* < 0.01 vs. IR + ghrelin.

Next, we investigated whether ghrelin influenced autophagy. Either ghrelin or the NOD2 antagonist was continuously administered after intestinal I/R injury. Intestinal epithelium tissues were obtained 0, 4, or 20 h after the treatment and analyzed using electron microscopy. As shown in Figure 2, the numbers of autophagosomes and autolysosomes increased 4 h and decreased 20 h after I/R. Moreover, ghrelin treatment obviously enhanced the numbers of autophagosomes and autolysosomes compared to those in the sham group (Figures 2 and 3). In addition, the numbers of autophagosomes and autolysosomes were remarkably lower in ghrelin + NOD2 antagonist rats than in ghrelin-treated rats (Figures 2 and 3). Our results revealed that the NOD2 antagonist suppressed the phagocytic functions of ghrelin in the intestinal epithelium of rats with I/R injury.

### 3.3 Ghrelin inhibited inflammatory responses after intestinal I/R injury

**Figure 4:**
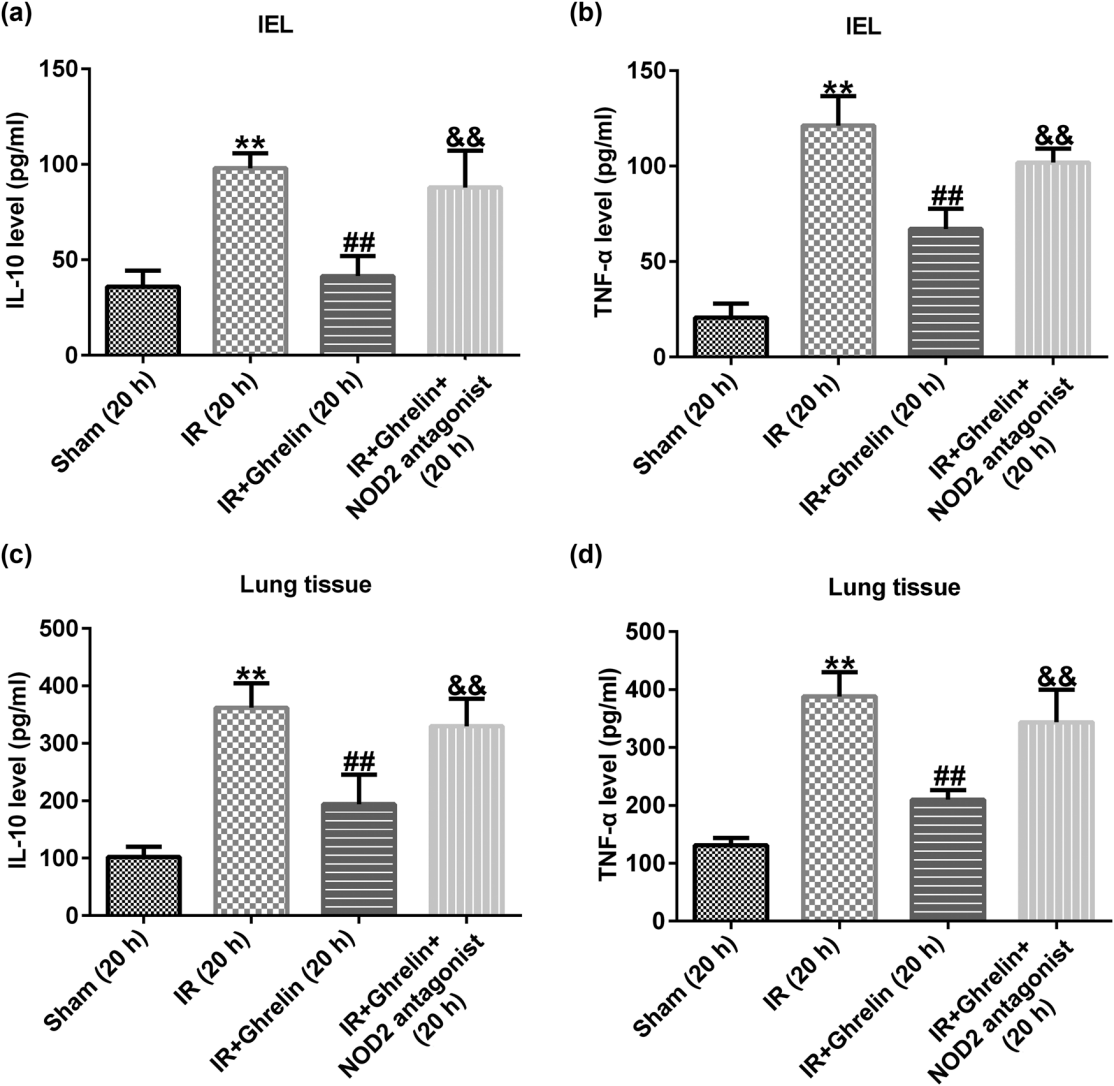
NOD2 antagonist reversed the effects of ghrelin on the release of inflammatory factors in IEL cells and lung tissue. Pro-inflammatory cytokine levels in IEL cells and lung tissue were evaluated using an ELISA. Changes were observed in the levels of IL-10 (a and c) and TNF-α (b and d) in IEL cells and lung tissue in the sham, I/R injury, ghrelin, and ghrelin and NOD2 antagonist groups after reperfusion. Data were expressed as mean ± SD, *n* = 6; **P < 0.01 vs sham; ^##^ *P* < 0.01 vs IR; and *P* < 0.01 vs IR + ghrelin.

Inflammatory responses were involved in the damage of organs after intestinal I/R. To explore whether the cytokines TNF-α and IL-10 were influenced by ghrelin treatment, we determined their levels using ELISA. We observed that the administration of ghrelin obviously inhibited the release of inflammatory factors in IEL cells, which increased in intestinal I/R injury models. However, the opposite results were observed in the ghrelin + NOD2 antagonist group (Figure 4a and b). In addition, we determined TNF-α and IL-10 production in the lungs using ELISA. We observed similar inhibition effects of ghrelin on the expression of the aforementioned cytokines in the pulmonary tissues (Figure 4c and d). Our findings suggested that the NOD2 antagonist reversed the effects of ghrelin on the production of inflammatory factors in IEL cells and lung tissues.

### 3.4 Ghrelin regulated IEL cell autophagy after I/R via the NOD2/Beclin-1 pathway

**Figure 5:**
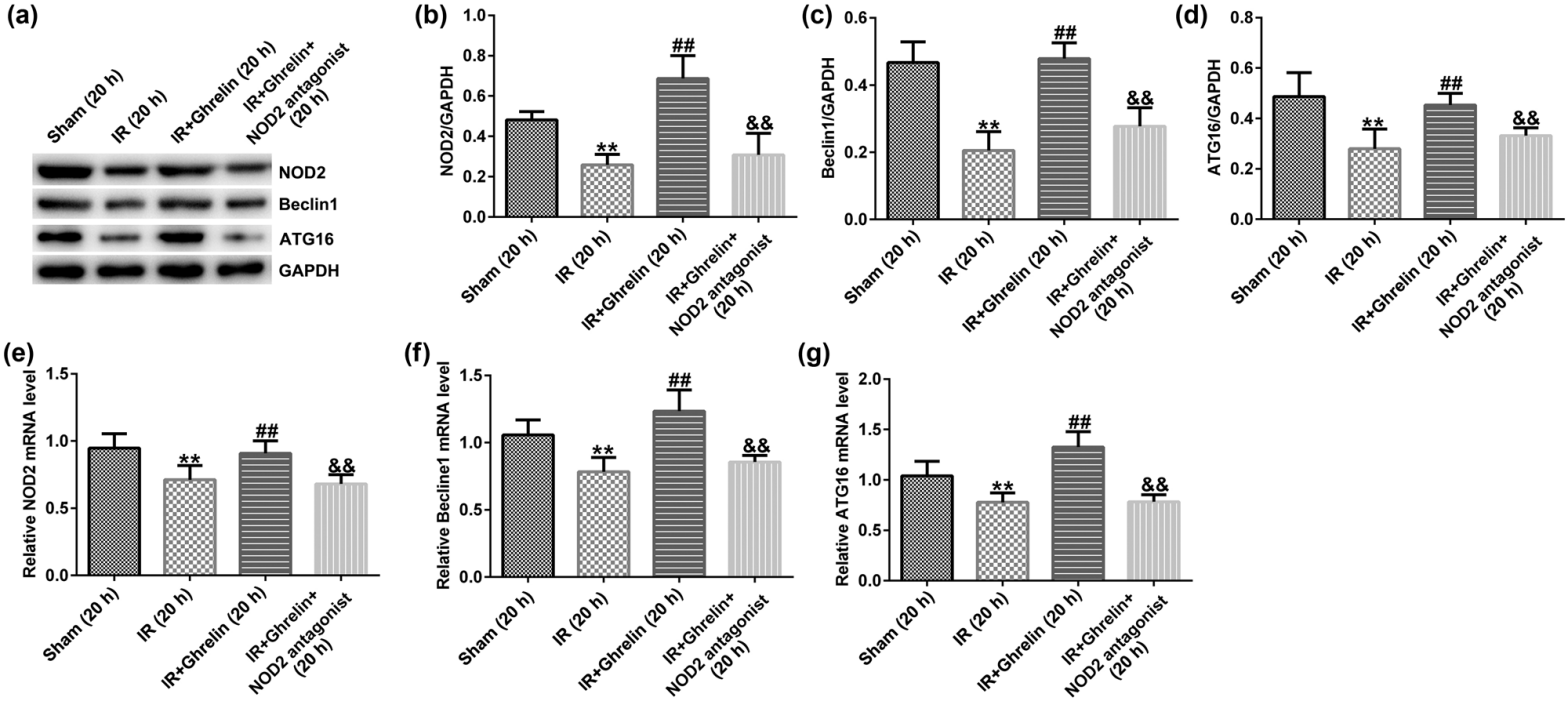
NOD2 antagonist reversed the effect of ghrelin on autophagy-associated proteins. (a–d) The protein levels of NOD2, Beclin-1, and ATG16 were determined using western blotting. (e–g) Detection of the NOD2, Beclin-1, and ATG16 mRNA expression levels in different groups (sham, I/R injury, ghrelin, or a combination of ghrelin and the NOD2 antagonist) after reperfusion. Data were expressed as mean ± SD, *n* = 6; **P < 0.01 vs sham; ^##^ *P* < 0.01 vs IR; and *P* < 0.01 vs IR + ghrelin.

To further investigate whether ghrelin regulated the autophagy of IEL cells after I/R via the NOD2/Beclin-1 pathway, we determined the expression of autophagy-associated proteins using qRT-PCR and western blot assays. Our results revealed that the levels of NOD2, ATG16, and Beclin-1 were upregulated after the ghrelin treatment, while the levels of NOD2, ATG16, and Beclin-1 were downregulated in the I/R + ghrelin + NOD2 antagonist group due to the action of NOD2 antagonist (Figure 5a‒g). Our findings indicated that ghrelin enhanced the autophagy of IEL cells by activating the NOD2/Beclin-1 pathway.

### 3.5 NOD2 antagonist reversed the protective effects of ghrelin on lung tissue damage

**Figure 6:**
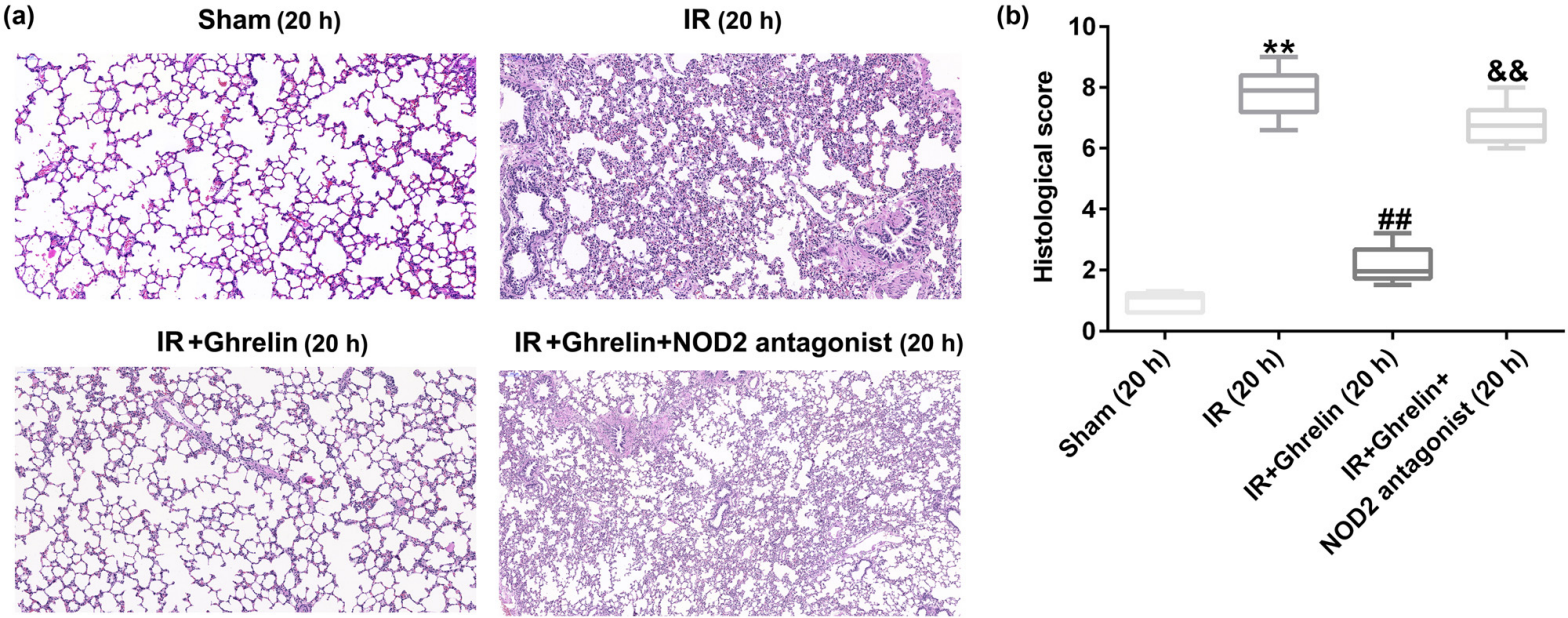
NOD2 antagonist reversed the effects of ghrelin on lung tissue damage. (a) Lung tissue samples from rats of different groups were stained with hematoxylin and eosin. (b) The histological score was determined. Data were expressed as mean ± SD, *n* = 6; **P < 0.01 vs sham; ^##^ *P* < 0.01 vs IR; and *P* < 0.01 vs IR + ghrelin.

To further explore the distant organ damage in rats of intestinal I/R injury, we determined the damage to the lung tissues in different groups. The hematoxylin and eosin staining results revealed that ghrelin relieved the degree of lung tissue damage, which was induced by I/R injury, while these protective effects were abolished by the NOD2 antagonist (Figure 6a). Meanwhile, compared with the sham group, the histological score in the IR group significantly enhanced. Compared with the IR group, the histological score in the IR + ghrelin group significantly reduced, while this reduction was significantly increased by NOD2 antagonist (Figure 6b). In summary, our findings verified the protective role of ghrelin in the intestinal I/R injury model by regulating autophagy disorder and inflammatory response via the NOD2/Beclin-1 pathway.

## 4 Discussion

Intestinal I/R injury is a serious, multifactorial complication in various pathophysiological and clinical conditions, such as sepsis, vascular surgery, and absorption dysfunctions [25,26]. Moreover, intestinal I/R injury may lead to autophagy dysfunction and the abnormal activation of IEL. The activation of immune cells, which lie adjacent to the surface area of endothelial cells, may lead to systemic inflammatory responses and severe tissue injury [27]. Previous studies confirmed that intracellular pattern recognition receptor NOD2 is involved in the regulation of autophagy and activation of immune cells, while the role of NOD2/Beclin-1 in intestinal I/R injury remains unknown. This study for the first time investigated the role of NOD2/Beclin-1 in intestinal I/R injury.

Zhang et al. confirmed that the plasma levels of ghrelin are obviously suppressed after intestinal I/R injury [28]. Ghrelin, a gastrointestinal hormone, plays pivotal roles both in the central and peripheral nervous systems [29]. Raghay et al. showed that ghrelin acts as an anti-inflammatory and protective factor in I/R injury [30]. Moreover, Wang et al. revealed that ghrelin protects the heart against I/R injury by inhibiting the TLR4/NLRP3 pathway [31]. However, the regulation of ghrelin in the NOD2/Beclin-1 pathway remains to be explored.

According to previous reports, we established an animal model of intestinal I/R injury in Sprague–Dawley rats using ghrelin or a NOD2 antagonist [32]. The animals were euthanized 4 or 20 h after the treatment, at which point we collected their small intestines, peripheral blood, and lungs. In this study, ghrelin relieved the I/R-induced destruction of the intestinal mucosa epithelium and lung tissues. Electron microscopy revealed that ghrelin enhanced autophagy in the intestinal epithelium of rats with I/R injury. Studies have reported that autophagy is involved in CLP-induced sepsis, and autophagy is upregulated in the early phase but downregulated later [23,33,34]. In this study, the findings for the first time indicated that the autophagy levels in IEL significantly increased at 4 h after intestinal I/R but decreased again at 20 h compared to 4 h after intestinal I/R, suggesting that autophagy might be upregulated in the early phase of intestinal I/R injury but downregulated later.

Our previous study demonstrated that ghrelin not only promotes autophagy-related genes, including Beclin-1 and Atg16, in septic intestinal epithelial cells, but also protects them by promoting autophagy [23]. In addition, ghrelin regulates the NOD2 mRNA levels and improves lung injury in septic rats, suggesting that ghrelin has a potential role in the regulation of NOD2-mediated autophagy [24]. Then, we investigated whether ghrelin can affect autophagy via the NOD2/Beclin-1 pathway and further affect the small intestine. In our study, we checked the levels of autophagy-associated proteins and genes using western blot and qRT-PCR analyses. Our findings suggested that ghrelin upregulated NOD2, Atg16, and Beclin-1 mRNA as well as protein expression levels, while the NOD2 antagonist produced the opposite results. Lucchi et al. revealed that the involvement of the ghrelin receptor antagonist enhanced pro-inflammatory factor release in healthy animals, promoted intestinal barrier dysfunction, and aggravated organ injury [35]. We isolated IEL cells from the small intestine of rats with I/R injury and determined the release of pro-inflammatory cytokines and autophagy-related protein expression in IEL cells. Our results indicated that the TNF-α and IL-10 levels in IELs were obviously enhanced after I/R; this increase may lead to IEL detachment from the epithelium. Meanwhile, the TNF-α and IL-10 levels decreased in the ghrelin-stimulated group, while the TNF-α and IL-10 levels increased in the I/R + ghrelin + NOD2 antagonist group due to the action of NOD2 antagonist. We obtained similar findings in the lung tissues, suggesting that ghrelin reduced excessive inflammation and organ injury through the NOD2/Beclin-1 pathway. Taken together, these findings indicate that ghrelin enhanced IEL autophagy and reduced excessive inflammation and organ injury by activating the NOD2/Beclin-1 pathway. However, this study still has some limitations. For example, how ghrelin acts on NOD2 remains to be further explored. In addition, the significance of the significant decrease in IEL autophagy levels at 20 h after intestinal I/R compared to 4 h after intestinal I/R remains to be explored. We will explore this more deeply in future research.

## 5 Conclusion

Our findings verified the protective role of ghrelin in an intestinal I/R injury model by regulating organ damage, autophagy disorders, and the inflammatory response via the NOD2/Beclin-1 pathway. These results provide a therapeutic basis for ghrelin in the treatment of intestinal I/R injury.

## Abbreviations

IEL: intestinal intraepithelial lymphocytes
I/R: ischemia–reperfusion
